# Therapeutic Effect of Cistanoside A on Bone Metabolism of Ovariectomized Mice

**DOI:** 10.3390/molecules22020197

**Published:** 2017-01-24

**Authors:** Xiaoxue Xu, Zhuanzhuan Zhang, Wenping Wang, Huiqin Yao, Xueqin Ma

**Affiliations:** 1 Department of Pharmaceutical Analysis, School of Pharmacy, Ningxia Medical University, 1160 Shenli Street, Yinchuan 750004, China; 2241823@sina.com (X.X.); 15109604869@163.com (Z.Z.); wangwenpingg@sina.com (W.W.); huiqin_yao@163.com (H.Y.); 2 Key Laboratory of Hui Ethnic Medicine Modernization, Ministry of Education, 1160 Shenli Street, Yinchuan 750004, China

**Keywords:** cistanoside A, ovariectomized mice, antiosteoporotic, TRAF6, RANKL

## Abstract

Cistanoside A (Cis A), an active phenylethanoid glycoside isolated from *Cistanche deserticola* Y. C. Ma, has received our attention because of its possible role in the treatment of osteoporosis. In the present study, we evaluated the effects of Cis A on an ovariectomized (OVX) mice model and investigated its underlying molecular mechanisms of action. After 12 weeks of orally-administrated intervention, Cis A (20, 40 and 80 mg/kg body weight/day) exhibited significant antiosteoporotic effects on OVX mice, evidenced by enhanced bone strength, bone mineral density and improved trabecular bone microarchitecture. Meanwhile, the activities of bone resorption markers, including tartrate-resistant acid phosphatase (TRAP), deoxypyridinoline (DPD) and cathepsin K, were decreased, and the bioactivity of bone formation marker alkaline phosphatase (ALP) was increased. Mechanistically, Cis A inhibited the expression of TNF-receptor associated factor 6 (TRAF6), an upstream molecule that is shared by both nuclear factor kappa-light chain enhancer of activated B cells (NF-κB) and phosphatidylinositol 3-kinase (PI3K)/Akt pathways and subsequently suppressed the levels of receptor activators of nuclear factor kappaB ligand (RANKL), downregulated the expression of NF-κB and upregulated osteoprotegerin (OPG), PI3K and Akt, which means Cis A possessed antiosteoporotic activity in ovariectomized mice via TRAF6-mediated NF-kappaB inactivation and PI3K/Akt activation. Put together, we present novel findings that Cis A, by downregulating TRAF6, coordinates the inhibition of NF-κB and stimulation of PI3K/Akt pathways to promote bone formation and prevent bone resorption. These data demonstrated the potential of Cis A as a promising agent for the treatment of osteoporosis disease.

## 1. Introduction

Osteoporosis, a systemic skeletal “silent killer”, has become a major health hazard afflicting over 2000 million people worldwide in recent years [1]. It is characterized by low bone mass density (BMD) and micro-architectural deterioration, which derive from an excess of bone resorption over bone formation and, finally, result in osteoporotic fracture [2,3]. Nowadays, identification of agents to block osteoclastic differentiation and resorption are the common and successful strategy for the development of therapeutic drugs for osteoporosis [4], and there are indeed many synthetic agents, including estradiol valerate and alendronate sodium, that could prevent and treat osteoporosis. However, the drugs for the diseases are far from ideal; some of these drugs could increase risk of endometrial and breast cancer and also have a degree of side effects, such as hypercalcemia, hypercalciuria, etc. [5], which limit their clinical applications. Therefore, for more than a millennium, traditional Chinese medicines (TCM), especially the edible TCM with the isolated bioactive compounds and fractions, have been extensively used safely and effectively in Asian countries to prevent and treat various diseases, including osteoporosis [6,7].

Osteoporosis is characterized as an obviously enhanced bone resorption due to increased osteoclastogenesis, and this process involves the commitment of hematopoietic monocytes into osteoclast precursors, which fuse to form multinucleated osteoclasts that target bone sites undergoing remodeling [4]. Receptor activator for nuclear factor-κB ligand (RANKL), a key factor that is secreted from osteoblasts, stimulates monocyte differentiation into osteoclasts [8,9]. The interaction of RANKL with its receptor RANK results in a cascade of intracellular events, including NF-κB, PI3K/Akt, calcium/calmodulin-dependent kinase by recruiting the adaptor signal protein TNF receptor-associated factor (TRAF6). As a result, a number of osteoclast-related marker genes, including TRAP, cathepsin K and DPD, are upregulated, and the process of bone resorption is accelerated.

Phenylethanoid glycosides are characterized by cinnamic acid and hydroxyl phenyl ethyl moieties that are attached to a β-glucopyranose (apiose, galactose, rhamnose, xylose, etc.) via a glycosidic bond, which are widely distributed in medicinal plants [10]. Cistanoside A (Cis A) is an active phenylethanoid glycoside in *Cistanche deserticola* Y. C. Ma. According to the record of Chinese pharmacopoeia, *C*. *deserticola* was traditional used to treat kidney-yin deficiency, muscle weakness, lumbar debility, etc., and phenylethanoid glycosides are the main bioactive constituents in this herb [11]. Based on the ‘kidney’ theory of TCM, the kidney could govern the bone system, which means the development and functions of the bones depend on the kidney-essence, and this kidney-essence can transform into bone marrow to nourish the bones, promote the growth and repair of the skeleton and strengthen the skeleton [12]. As *C*. *deserticola* could strengthen the kidney, we supposed that Cis A could prevent and treat osteoporosis. The present study was therefore designed to validate the potential of Cis A in preventing osteoporosis by using an ovariectomized mice model, and bone formation and resorption markers, as well as the related potential mechanisms were determined to estimate the antiosteoporotic bioactivity of this agent.

## 2. Results and Discussion

### 2.1. Results

#### 2.1.1. Effects of Cis A on Bone Three-Point Bending Testing

**Figure 1:**
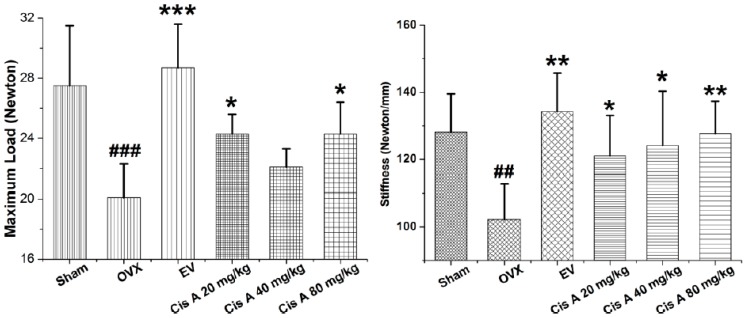
Effects of cistanoside A (Cis A) and estradiol valerate (EV) on bone strength analyzed by three-point bending of the femur of ovariectomized (OVX) mice (*n* = 4); all values are expressed as the mean ± SD. * *p* < 0.05, ** *p* < 0.01, *** *p* < 0.001 as compared to the OVX group; ^##^ *p* < 0.01, ^###^ *p* < 0.001 as compared to the sham group.

To analyze whether treatment with Cis A makes the bone stronger, we subjected femurs to the three-point bending test. As shown in Figure 1, maximal load applied when the bone breaks was 21.5% and 22.0% higher in the animals treated with 20 mg/kg and 80 mg/kg Cis A, respectively, compared to animals in the ovariectomized (OVX) group (*p* < 0.05). Meanwhile, treatment with Cis A also enhanced the bone stiffness; all of the Cis A-treated mice showed significantly increased stiffness with data of 121.0 ± 12.1 (*p* < 0.05), 124.1 ± 16.2 (*p* < 0.05) and 127.7 ± 9.6 (*p* < 0.01), respectively, when compared to 102.2 ± 10.7 of the OVX mice. The results indicate that the increased bone strength in the OVX mice treated with Cis A was due to an increased amount of bone and an enhancement of bone quality.

#### 2.1.2. Effects of Cis A on Bone Microarchitecture

**Figure 2:**
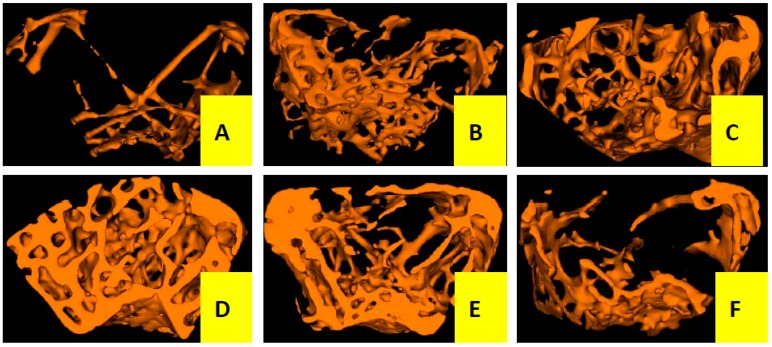
Micro-CT scan of right femur of mice in each group (*n* = 4); (**A**) OVX group; (**B**) sham group; (**C**) EV group; (**D**) Cis A 80 mg/kg group; (**E**) Cis A 40 mg/kg group; (**F**) Cis A 20 mg/kg group. The OVX mice showed a notable decrease in the trabecular area and trabecular number. Cis A treatment and EV treatment partially prevented OVX-induced bone loss and significantly improved the microarchitecture after the 12-week intervention.

Three-dimensional trabecular bone microarchitecture of mice measured by micro-CT (Figure 2 and Table 1) intuitively shows that the mice in the OVX group presented a notable reduction in the trabecular area and trabecular number when compared to the sham group, indicating that ovariectomy could induced a notably decrease in bone mass density (BMD, −46%), bone mineral content (BMC, −66%), tissue mineral content (TMC, −85%), bone volume fraction (BVF, −82%), trabecular number (Tb.N, −76%) and an increase in trabecular separation (Tb.Sp, +80%) without any modification in total tissue mineral density (TMD) and trabecular thickness (Tb.Th) after the operation of 12 weeks. However, the OVX mice treated with Cis A resulted in dose-dependently increased BMD (+43%~57%), BMC (+65%~73%), TMC (+83%~90%), BVF (+80%~88%), greater reduction in Tb.Sp (−79%~88%) and further enhanced Tb.N (+73%~82%) compared to the OVX group. TMD seemed not to be influenced by the ovariectomy, but was significantly increased by treatment with estradiol valerate (EV).

#### 2.1.3. Effects of Cis A on Both the Bone Formation and Resorption Markers

**Figure 3:**
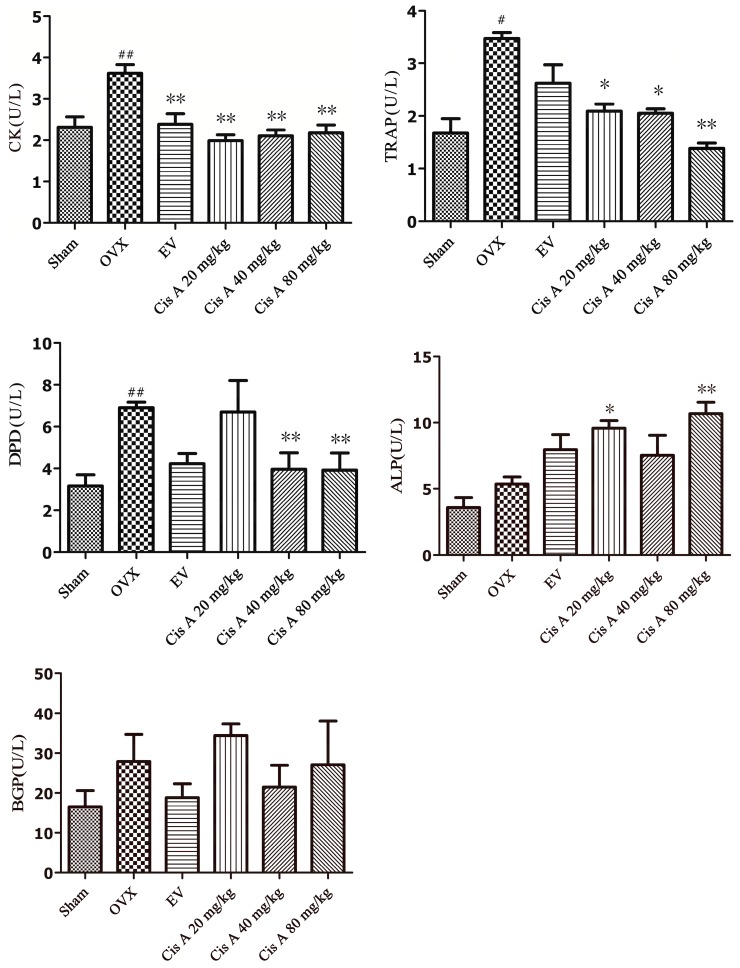
Effects of Cis A and EV on serum cathepsin K, DPD, TRAP, ALP and bone Gla-protein (BGP) activities of OVX mice (*n* = 8~10); all values are expressed as the mean ± SD. * *p* < 0.05, ** *p* < 0.01 as compared to the OVX group; ^#^ *p* < 0.05, ^##^ *p* < 0.01 as compared to the sham group.

The effects of Cis A on bone resorption markers, including TRAP, DPD, cathepsin K and bone formation index ALP and bone Gla-protein (BGP), are shown in Figure 3. After 12 weeks of ovariectomy operation, the activities of TRAP, DPD and cathepsin K in the OVX group were significantly increased, especially DPD, which increased by nearly 55.6%; TRAP and cathepsin K were enhanced by 43.5% and 38.1%, respectively, as compared to the sham group. Cis A, administered orally for 12 weeks, demonstrated a notably potential in preventing all of the above-mentioned bone resorption markers, especially the high dosage (80 mg/kg) exhibiting a significant effect on suppressing the activities of DPD by 45.0%, TRAP by 49.0% and cathepsin K by 44.0%, respectively (*p* < 0.01), as compared to the OVX group (Figure 3). Although an increasing trend of ALP and BGP activities was demonstrated in the OVX group, no statistically-significant changes were observed. However, a significant improvement of ALP activity was observed in low and high Cis A-treated groups as compared to the sham group (*p* < 0.01).

#### 2.1.4. Effects of Cis A on Protein Expression Levels of TRAF6, NF-κB PI3K, Akt, OPG and RANKL

**Figure 4:**
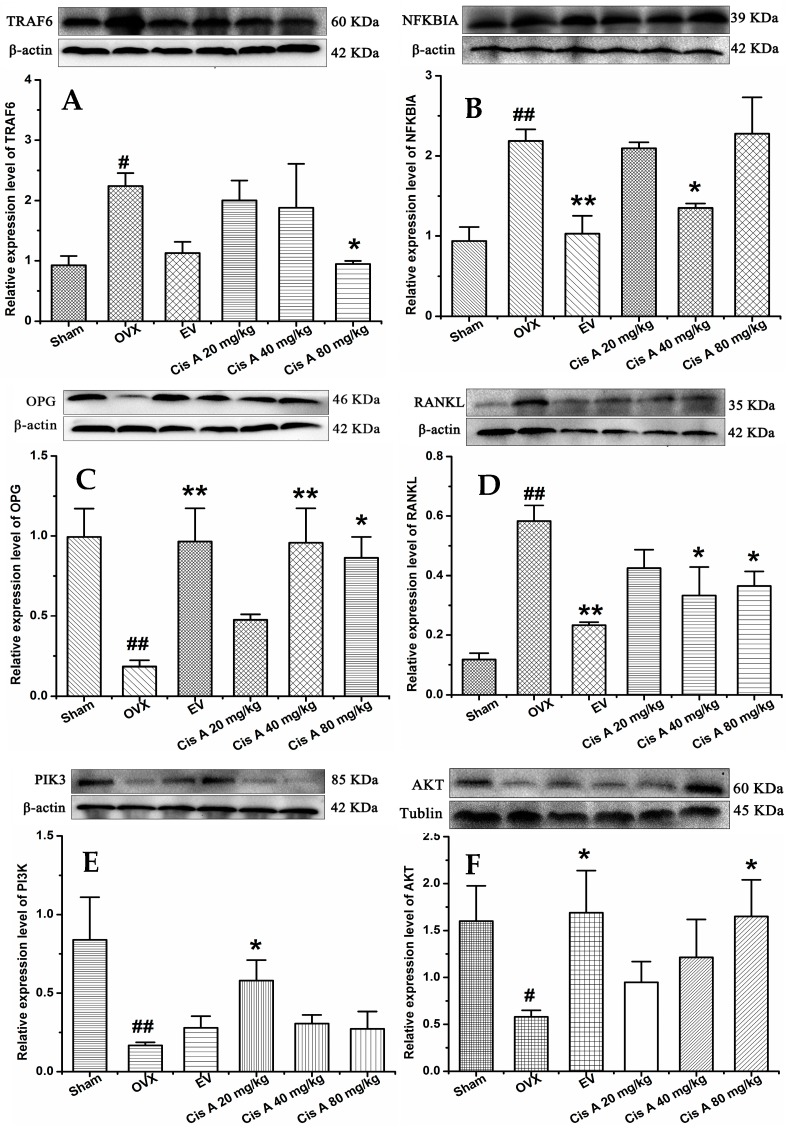
Effects of Cis A on the expression of TRAF6 (**A**), NF-κB (**B**), OPG (**C**), RANKL (**D**), PI3K (**E**) and Akt (**F**); β-actin or tubulin is shown as the loading control, and quantitative data of every signal protein are shown as percentages of the value of control mice. Values are presented as the mean ± SD (*n* = 5). * *p* < 0.05, ** *p* < 0.01 compared with the sham group: ^#^ *p* < 0.05, ^##^ *p* < 0.01 compared with the OVX group.

Western blot analysis revealed that compared to the sham group, the protein levels of TRAF6, NF-κB and RANKL in the OVX group were significantly increased (*p* < 0.05), while OPG, PI3K and Akt were significantly decreased (Figure 4). Cis A (20 mg/kg or 80 mg/kg) significantly downregulated TRAF6 expression (*p* < 0.05), followed by the RANKL expression being decreased and OPG increased, which means the OPG/RANKL ratio was upregulated. Consequently, the signaling cascades of NF-κB were downregulated and of PI3K/Akt were upregulated by Cis A treatment (*p* < 0.05).

### 2.2. Discussion

Given the limitations of current therapeutic options for osteoporosis disease, there is a need for alternatives from food or natural edible medicinal plants. As a part of our ongoing effort to discover effective antiosteoporotic agents from TCM, we found a series of extracts, fractions and compounds that possess the effect of antiosteoporotic properties [13,14]. *Cistanche deserticola* is an important classic TCM, which was found to possess a favorable safety profile [15] and broad medicinal functions for the treatment of kidney deficiency, etc. [16]. According to the theory of TCM, TCM that possess the effect of invigorating kidney were usually used to treat osteoporosis; phenylethanoid glycosides are the main bioactive constituents in this herb, thus implying that phenylethanoid glycosides contained in *C*. *deserticola* may possess an antiosteoporotic property. It was proven that *C*. *deserticola* extract could significantly inhibit the reduction in BMD and prevent the deterioration of trabecular microarchitecture caused by OVX [17]. In the in vitro experiment, it also significantly increased ALP, bone morphogenetic protein-2 and osteopontin mRNA, as well as bone mineralization of cultured osteoblasts [18]. Echinacoside, a main bioactive component in *C*. *deserticola* officially recorded in the Chinese pharmacopoeia [11], exhibited antiosteoporotic activity with a high dosage of 30~270 mg/kg body weight/day [19], and further in vitro results showed that it could promote bone regeneration by increasing the OPG/RANKL ration in MC3T3-E1 Subclone 14 cells [20]. Cis A was one of the phenylethanol glycosides isolated from *C*. *deserticola*, and several reports revealed that this compound possessed antioxidative activity [21] and anti-inflammatory properties [22,23]. A recent published paper discovered that Cis A exhibited protective activities on both CCl_4_ and alcohol-induced hepatotoxicity in mice, and it also showed a protective property on ethanol-induced damage in primary cultured mouse hepatocytes in vitro [24]. In our present study, the results showed that Cis A possessed antiosteoporotic activity at a low dosage (20~80 mg/kg body weight/day) by using an ovariectomized mice model, and this bioactivity was exerted by downregulating the level of TRAF6, suppressing the expression of RANKL and NF-κB and stimulating OPG, PI3K and Akt, which means the therapeutic effect of Cis A in OVX mice was through the mechanism of TRAF6-mediated NF-kappaB inactivation and PI3K/Akt activation.

It is well known that ovariectomy can cause osteoporosis with an obvious decrease in BMD, biomechanical strength, bone quality and trabecular bone microarchitecture, and the above changes are in part due to estrogen deficiency [25]. Now, in the present in vivo experiment, our study clearly demonstrates that ovariectomy-induced osteoporosis resulted in a significant reduction in biomechanical strength and trabecular structural parameters, including BMD, BMC, TMC and Tb.N, and increasing Tb.Sp; and treatment of Cis A significantly improved bone mechanical properties including maximum load and stiffness, enhanced the BMD and improved most of the bone trabecular microarchitecture structural parameters in comparison with the mice in the OVX group, indicating that Cis A was effective in ameliorating bone quality and trabecular microarchitecture in OVX mice.

Besides the total BMD, the three-point bending test and trabecular bone microarchitecture measurement could directly diagnose osteoporosis, the bone formation markers, including ALP and BGP, and the bone resorption index, including TRAP, DPD and cathepsin K, was also employed to elucidate the related antiosteoporotic mechanisms of Cis A. In our study, ALP activity in the OVX group mice demonstrated a non-significant increasing trend, which indicating an increased rate of bone turnover [26,27] in postmenopausal osteoporosis; the high (80 mg/kg body weight/day) and low (20 mg/kg body weight/day) dosage of Cis A treatments showed significant enhancement on ALP activity compared to the sham group, whereas BGP activity seemed not to be influenced by the ovariectomy in all treated groups; TRAP, DPD and cathepsin K were significantly increased in the OVX group, and Cis A administration notably decreased all three bone resorption markers. The above data implied that Cis A possessed potential antiosteoporotic activity, and this effect was exerted by the regulation of bone metabolism, including both suppressing bone resorption and increasing bone formation.

The coordination between osteoblast and osteoclast is a critical factor in the maintenance of skeletal integrity. Osteoclasts, expressing TRAP, attach to the surface of bone through the formation of actin-bound sealing zones, within which proteolytic enzymes, such as cathepsin K, are released, leading to the formation of resorption pits. The modulation of osteoclastogenesis by immature cells of the osteoblastic lineage is mediated by RANKL and OPG [28]. OPG is a decoy receptor that inhibits RANKL activation of osteoclastogenesis, thereby decreasing bone resorption. RANKL, which provides an important signal to osteoclast progenitors, is a membrane-bound molecule of the tumor necrosis factor ligand family that promotes the formation of osteoclasts. The ratio of OPG/RANKL expression is believed to be a key parameter of osteoclastogenic activity, and signaling cascades activated by RANKL include the NF-κB and PI3K pathways [29]. The importance of the NF-κB pathway to osteoclastogenesis is demonstrated by the fact that deletion of NF-κB in mice resulted in the absence of mature osteoclasts [30]. TRAF6 was proven as a promising target for novel anti-osteoporotic drugs. TRAF6-deficient mice exhibiting defective osteoclastogenesis and severe osteopetrosis thus demonstrated the importance of TRAF6 in bone metabolism. Emerging evidence points to a critical regulatory function for TRAF6 in RANKL/RANK-mediated signaling cascades [4,31]. The data of the current study indicated that Cis A treatment on the OVX mice resulted in the downregulation of TRAF6 protein expression levels, decreased RANKL and increased OPG expressions and thereby prevented RANKL activation of downstream NF-κB and activating the PI3K/Akt signal pathways, suggesting that Cis A inhibits osteoclast differentiation by TRAF6-mediated NF-kappaB inactivation and PI3K/Akt activation and increasing the OPG/RANKL ratio, subsequently inhibiting osteoclastogenesis and promoting bone formation.

## 3. Materials and Methods

### 3.1. Plant Materials, Preparation and Determination

**Figure 5:**
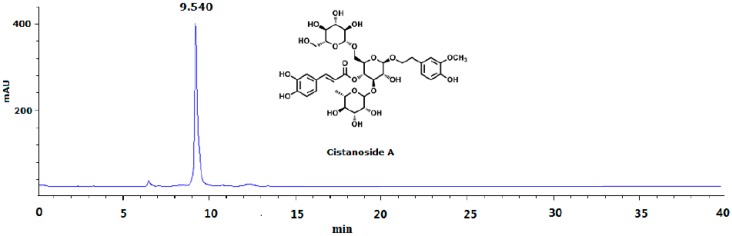
HPLC analysis of cistanoside A (Cis A). Cis A was dissolved in methanol for HPLC analysis, which was carried out on an Agilent 1220 HPLC instrument by using the TSK-GEL C18 column (4.6 μm × 250 mm; 5 μm) at room temperature. The mobile phase gradient elution was as follows: Solvents A (water containing 0.5% acetic acid) and B (acetonitrile): 0–10 min, 83%–80% A; 10–30 min, 80%–75% A; 30–40 min, 75%–70% A. The flow rate was 1.0 mL/min; the detection wavelength was 333 nm; the sample was found to have 95.5% purity by HPLC analysis.

Stems of *Cistanche deserticola* Y. C. Ma, identified by Prof. Xue-yan Fu of the department of pharmacy, Ningxia Medical University, were collected in September of 2015 in Yongning County, Ningxia province, China. A voucher specimen (#20150901) was conserved in the herbarium of the pharmacy department. A total of 30.0 kg of air-dried stems of *C. deserticola* were reflux-extracted with 70% ethanol (180 L, 3 × 2 h) 3 times. The filtrates were combined and subjected to a macroporous resin (AB-8) column with a stepwise gradient solvent system of increasing ethanol in water (0%, 20% and 60%, each 60 L) to give three fractions, then 60% of the fraction was further purified by Sephadex LH-20 (Pharmacia Biotech Co., Uppsala, Sweden) and semi-preparative high-performance liquid chromatography (Agilent 1100, Agilent Technologies, Waldbronn, Germany) to obtain pale yellow cistanoside A 4.5 g (Cis A; the yield was 0.015%). The structure was elucidated mainly by NMR (Bruker Avance, 400 MHz, Germany) and MS spectra (Agilent 6540, Agilent Technologies) data analyses, which was consistent with the reported data [19]. Its purity was determined by the HPLC area normalization method (Figure 5). In animal experiments, based on our preliminary experiment, the dosage of Cis A was 20–80 mg/kg/day.

### 3.2. Chemicals and Solvents

The following were used: total protein extraction kit and BCA protein quantization kits (Ken Gen Biotech. Co. Ltd, Nanjing, China), primary antibodies of TRAF6, NF-κB, PI3K, Akt, RANKL, OPG, β-actin and tubulin (Cell Signaling Technology, Beverly, MA, USA), secondary antibodies of horseradish peroxidase-conjugated goat anti-rabbit IgG (ZSGB-BIO, Beijing, China), estradiol valerate (1 mg, Delpharm Lille S.A.S, Paris, France), bone Gla-protein and deoxypyridinoline crosslinks ELISA kits (Xinyu Biological Engineering Co. Ltd., Shanghai, China) and the cathepsin K ELISA kit (Biovision, Mountain View, CA, USA). All other chemicals and biochemical agents used were of AR analytical grade reagent.

### 3.3. Animal Experiments

Sixty female Kunming strain mice, aged 8 weeks with a body weight of 26.0 ± 1.62 g, were purchased from Ningxia Medical University, and 10 mice were kept in one cage with a standard laboratory diet and tap water under an air-controlled condition (24.0 ± 0.5 °C, 45%–50% humidity and 12 h/12 h light-dark illumination cycles). After being acclimated with 1 week, surgery was done under an aseptic condition, and all of the mice were anesthetized with chloral hydrate (100 mg/kg, i.p.); the mice that were subject to surgery exposure without removing the ovaries are the sham group (*n* = 10); the others were subjected to bilateral ovariectomy as the OVX groups (*n* = 50). The above animal experiments were carried out according to the guide for the care and use of laboratory animals and were approved by the Bioethics Committee of the Ningxia Medical University.

After another 1-week recovery period, the above 50 OVX mice were randomly and equally divided into 5 groups: orally treated with estradiol valerate (1 mg/kg/day) as the positive control (EV); with 20, 40 and 80 mg/kg/day of Cis A as the low, moderate and high dosage groups, respectively; with the vehicle (0.5% Carboxymethylcellulose()-Na) as the model group (OVX). All of the mice were orally administered with an equal volume of 1 mL/100 g body weight vehicle, EV or Cis A, which started on the second week after operation and lasted for 12 weeks. Body weight was measured biweekly, with the dose adjusted accordingly. After the last oral administration, blood samples were withdrawn from the femoral artery of anesthetized mice, allowed to clot and centrifuged at 3000× *g* for 10 min to afford serum; the right femora and tibia were dissected. All of the samples were stored at −80 °C for further evaluation of bone microarchitecture and osteoporotic-related serum biochemical parameters.

### 3.4. Three-Point Bending Testing

The biomechanical property of the right femur of mouse was determined by using a three-point bending arrangement. The bending jig has a V-shaped support to secure the distal and the proximal ends of the femur during testing. The femur was positioned horizontally with the anterior side upwards, and the load was applied downwards in the center of the mid-femur. The distance between the supports was 8 mm. The bones were loaded at a constant displacement rate of 0.02 mm/s until failure using a BOSE Testing Machine (3220) (Bose Corporation, Endura TEC Systems Group, Minnetonka, MN, USA). The load-displacement curves were obtained and used for the calculation of the structural and material properties, including maximum load, peak load, elastic modulus, flexural strength, etc.

### 3.5. Micro-Computed Tomography Analysis

The right femurs of all of the mice were cleaned of adhering soft tissues, and the trabecular bone microarchitecture of the above right distal femoral metaphysic was determined by a micro-CT scanner (eXplore Locus SP, GE Healthcare, London, ON, Canada) with the isotropic resolution set to 8 μm in all three spatial dimensions. The region of interest (ROI) was selected by using the same coordinates in the growth plate of the femur. The bone morphometric parameters, including BMD, bone mineral content (BMC), tissue mineral content (TMC), tissue mineral density (TMD), bone volume fraction (BVF), trabecular separation (Tb.Sp), trabecular number (Tb.N) and trabecular thickness (Tb.Th), were evaluated through analyzing the ROI by using ABA bone analysis software.

### 3.6. Biochemical Parameters’ Analysis

The activity of serum alkaline phosphatase (ALP) was measured on an automatic analyzer (Ciba-Corning 550 Diagnotics, Corp., Oberlin, OH, USA)); the levels of serum DPD, BGP and cathepsin K were determined by corresponding reagent kits, and tartrate-resistant acid phosphatase (TRAP) was analyzed according to the related literature [32].

### 3.7. Western Blot Analysis

Protein expression levels of TRAF6, NF-κB PI3K, Akt, OPG and RANKL in different groups were detected by Western blot analysis. Briefly, protein was extracted from the right tibia of mice using a lysis buffer containing 0.5 mM phenylmethylsulfonyl fluoride, protease and phosphatase inhibitors. Lysates were centrifuged for 10 min at 12,000× *g* to obtain the supernatants for further analysis. The protein concentration of the lysates was measured using a bicinchoninic acid quantification assay. Proteins (50–100 µg) were separated using 10% SDS-PAGE, transferred to a polyvinylidene difluoride membrane and incubated with monoclonal primary antibodies targeting TRAF6, NF-κB RANKL, PI3K, Akt, OPG and β-actin (1:500). The same membrane was stripped and reprobed, with chemiluminescent signals detected by the Image Lab software. β-actin or tubulin served as an internal control.

### 3.8. Statistical Analysis

All of the data obtained from animal experiments were analyzed by using one-way analysis of variance with Dennett’s test by SPSS (Version 22.0, IBM SPSS Statistics, IBM Corp., Armonk, New York, NY, USA) software; a value of *p* < 0.05 was considered statistically significant, and all of the results were presented as the mean ± SD.

## 4. Conclusions

In summary, Cis A, an active phenylethanoid glycosides isolated from *C*. *deserticola*, exhibited a significant antiosteoporotic activity on OVX mice, and the molecular mechanism may be related to TRAF6-mediated NF-kappaB inactivation and PI3K/Akt activation, thus likely a promising agent for the treatment of osteoporosis disease.

## Figures and Tables

**Table 1:** Effects of Cis A and EV on the microarchitecture of right distal femurs of OVX mice (*n* = 4).

Group	BMD (mg/cc)	BMC (mg)	TMC (mg)	TMD (mg/cc)	BVF	Tb.Sp (mm)	Tb.N (1/mm)	Tb.Th (mm)
Sham	719 ± 79	2.07 ± 0.25	1.10 ± 0.35	1780 ± 64	0.22 ± 0.07	0.26 ± 0.11	3.34 ± 0.91	0.06 ± 0.01
OVX	391 ± 74 ^#^	0.70 ± 0.43 ^##^	0.17 ± 0.19 ^#^	1717 ± 208	0.04 ± 0.03 ^#^	1.29 ± 0.44 ^###^	0.81 ± 0.27 ^###^	0.04 ± 0.01
Cis A 80 mg/kg	915 ± 298 **	2.64 ± 0.88 ***	1.75 ± 0.82 **	1773 ± 125	0.34 ± 0.13 ***	0.15 ± 0.05 ***	4.42 ± 0.62 ***	0.07 ± 0.02 *
Cis A 40 mg/kg	732 ± 32 *	2.00 ± 0.13 *	1.08 ± 0.06 *	1791 ± 80	0.22 ± 0.01 *	0.24 ± 0.01 ***	3.38 ± 0.22 ***	0.06 ± 0.01
Cis A 20 mg/kg	689 ± 71	1.98 ± 0.20 *	1.00 ± 0.13	1708 ± 35	0.20 ± 0.03 *	0.27 ± 0.04 ***	3.02 ± 0.29 **	0.07 ± 0.01
EV	1057 ± 335 ***	3.03 ± 0.95 ***	2.08 ± 0.90 ***	2090 ± 299 *	0.34 ± 0.15 ***	0.22 ± 0.13 ***	3.55 ± 1.38 ***	0.10 ± 0.03 **

All values are expressed as the mean ± SD, * *p* < 0.05, ** *p* < 0.01, *** *p* < 0.001 as compared to the OVX group; ^#^ *p* < 0.05, ^##^ *p* < 0.01, ^###^ *p* < 0.001 as compared to the sham group; bone mass density (BMD), bone mineral content (BMC), tissue mineral content (TMC), tissue mineral density (TMD), bone volume fraction (BVF), trabecular separation (Tb.Sp), trabecular number (Tb.N) and trabecular thickness (Tb.Th).
